# Using an innovative web-based task management application to improve medical handovers at an in-patient psychiatric hospital

**DOI:** 10.1192/j.eurpsy.2025.319

**Published:** 2025-08-26

**Authors:** H. Hamilton, K. Laffan

**Affiliations:** 1 Psychiatry, South London and Maudsley NHS Trust, London, United Kingdom

## Abstract

**Introduction:**

Safe and effective handovers are crucial for patient safety in psychiatric hospitals and a digital record helps to prevent information loss and allow auditing. Trainees at our institution raised concerns about the lack of a reliable, traceable digital record for out-of-hours handovers. The pre-existing system of handwritten notes and emails was error-prone, cumbersome and untraceable. Therefore, a more efficient digital system was needed to match the demands of a busy in-patient psychiatric hospital.

**Objectives:**

To improve the safety and quality of medical handovers in our hospital.

**Methods:**

A baseline survey was sent to resident doctors at The Maudsley Hospital, London, to gather feedback on handover practices. The Microsoft Planner application within Teams was introduced, providing a secure, live digital handover record accessible with two-factor authentication. It allows multiple users to edit tasks simultaneously and tracks all updates with clinician specific time stamps. Changes were communicated and feedback gathered through monthly meetings, emails, and encrypted messaging apps. After 12 weeks, we sent a follow-up survey and made further adjustments based on the feedback.

**Results:**

Nine doctors responded to the baseline survey, giving an average score of 2.5 out of 5 for quality and 2.4 out of 5 for safety. Forty-six percent struggled to prioritize tasks during on-call shifts, 54.6% found it hard to track task progress, and 18.2% reported frequent task delays or omissions. After the changes, nine doctors responded with improved scores of 4.6 out of 5 for quality and 4.2 out of 5 for safety. Seventy-eight percent rarely had difficulty prioritizing tasks, 66.7% found it easy to track progress, and 87.5% reported that tasks were rarely or never missed. Feedback highlighted that use of the platform varied amongst clinicians and access issues for emergency locum doctors, leading to the creation of a trust-wide protocol to standardise the use of this technology and respond to access issues.

**Conclusions:**

A web-based task management platform was introduced in a large in-patient psychiatric hospital with significant improvements seen in clinician-rated quality and safety of medical handovers. This application is now commonly used across South London and Maudsley NHS Trust, the largest mental health trust in the United Kingdom. Over 450 NHS organizations have access to the Microsoft 365 and could therefore use this innovative technology to improve their clinical handovers. Given Microsoft’s global use, this could very likely be used throughout European mental health organisations.

**Disclosure of Interest:**

None Declared

